# Construct validity of script concordance testing: progression of scores from novices to experienced clinicians

**DOI:** 10.5116/ijme.5d76.1eee

**Published:** 2019-09-20

**Authors:** Michael Siu Hong Wan, Elina Tor, Judith N. Hudson

**Affiliations:** 1 School of Medicine, University of Notre Dame, Australia; 2 Faculty of Health and Medical Sciences, University of Adelaide, Australia

**Keywords:** Script Concordance Testing, validity, assessment, clinical reasoning

## Abstract

**Objectives:**

To investigate the construct validity of Script Concordance Testing (SCT) scores as a measure of the clinical reasoning ability of medical students and practising General Practitioners with different levels of clinical experience.

**Methods:**

Part I involved a cross-sectional study, where 105 medical students, 19 junior registrars and 13 experienced General Practitioners completed the same set of SCT questions, and their mean scores were compared using one-way ANOVA. In Part II, pooled and matched SCT scores for 5 cohorts of students (2012 to 2017) in Year 3 (N=584) and Year 4 (N=598) were retrospectively analysed for evidence of significant progression.

**Results:**

A significant main effect of clinical experience was observed [F_(2, 136)_=6.215, p=0.003]. The mean SCT score for General Practitioners (M=70.39, SD=4.41, N=13) was significantly higher (p=0.011) than that of students (M = 64.90, SD = 6.30, N=105). Year 4 students (M=68.90, SD= 7.79, N=584) scored a significantly higher mean score [t_(552)_=12.78, p<0.001] than Year 3 students (M = 64.03, SD=7.98, N=598).

**Conclusions:**

The findings that candidate scores increased with increasing level of clinical experience add to current evidence in the international literature in support of the construct validity of Script Concordance Testing. Prospective longitudinal studies with larger sample sizes are recommended to further test and build confidence in the construct validity of SCT scores.

## Introduction

Since 2009, Script Concordance Testing (SCT) has been used to assess higher-order clinical reasoning and data interpretation skills in the context of uncertainty, at both undergraduate and postgraduate medical education levels.^1^ It was designed to probe one key signpost along an accepted theoretical pathway of clinical reasoning under uncertainty.^2^ In each SCT, candidates are presented with a clinical scenario, followed by a new piece of information. The candidates are then asked to assess whether this additional piece of information increases or decreases the probability of the suggested provisional diagnosis or increases or decreases the appropriateness of a proposed investigation or management option. In the classical scoring of SCT, the candidate’s decision is compared to that of a reference panel of experts in the field and a weighted partial scoring system with a 5-point Likert scale is applied.^3^ Since its development, the SCT format has been used in assessment across many medical disciplines, including Medicine, Surgery, Psychiatry, Paediatrics, Dentistry and more recently, Medical Ethics.^4^^-^^12^

As for all educational assessments, SCT use as summative assessment in Medicine requires evidence to support the appropriateness and meaningfulness of interpretation and use of the results.^13^ Over the past few years a number of studies in the international literature have addressed some issues on the validity of SCT scores.^9^^,^^14^ However, there is a relative paucity of evidence demonstrating that SCT scores are a measure that can discriminate between the reasoning skills of medical practitioners at different stages in their medical career – i.e. from medical students, to junior doctors, to experienced doctors. This is an important piece of evidence for the overall construct validity of SCT scores, an issue which this study aims to address. In general, construct validity is the degree to which an instrument measures the construct it is intended to measure.^15^^,^^16^ In the context of Script Concordance Testing, according to key developers of this assessment format, the construct validity of scores from script concordance testing depends on the inference that candidates with more evolved illness scripts interpret data and make judgments in uncertain situations that increasingly concord with those of experienced clinicians given the same clinical scenarios.^3^ The tendency for SCT scores to consistently increase with increasing level of training has been reported as empirical evidence supporting the validity of this inference.^17^

The progression of clinical reasoning capability, as measured by SCT in post-graduate medical education settings, has been reported in previous studies. In 2009 Lubarsky showed that Neurology trainees’ SCT scores improved as they progressed through the post-graduate training program. This evidence of progression of SCT scores supported the construct validity of SCT in this setting.^1^ There is also evidence of progression of clinical reasoning during residency emergency training in Paediatrics.^14^ Kazour examined interns (junior doctors in the first post-graduate year) using a set of 100 SCT questions in Psychiatry and found significant improvement in the interns' scores between the beginning and the end of their rotation.^8^ A further study used SCT scenarios to assess the reasoning skills of paediatric residents and neonatal-perinatal medicine fellows (qualified specialists), and reported a significant difference between all training levels from Post-graduate Year 1 (PGY-1) to PGY-3 and between PGY-3 and fellows, with improvement of scores observed for each progressive level of medical training.^18^ More recently, Subra administered an SCT assessment to post-graduate students in general practice and showed progression of clinical reasoning throughout the 3 years of training pathway especially in the first 18-months.^19^ However, there is an apparent gap in the literature, specifically in relation to empirical evidence of progression in clinical reasoning skills for medical students in undergraduate medical education. Furthermore, studies comparing the clinical reasoning capability of medical students and practising clinicians, using the same set of SCT items, are lacking. This study aimed to address these gaps by seeking evidence of progression of medical students’ SCT scores through the two senior clinical years, and evidence of higher scores for experienced clinicians and post-graduate trainees when compared with those of senior medical students (novices), on the same set of SCT questions. Progression in performance on SCTs, i.e. tendency for SCT scores to consistently increase with increasing level of training and experience, should provide further support for the hypothesis that SCT scores are a valid measure of clinical reasoning ability in Medicine.

The setting for the current study was a medical school in Australia with a four-year graduate-entry medical program. The School has been using SCT questions in Year 3 and Year 4 summative assessments of the program since 2010. The aim of the study was to investigate the construct validity of SCT scores as a measure of clinical reasoning ability of senior medical students and practising clinicians of differing experience in general medical practice (family medicine). Specifically, this study sought to test the following hypotheses for the construct validity of SCT scores:

1.     There is a significant progression in SCT scores from senior medical students, junior registrars, to experienced general practitioners (GPs), using the same set of SCT questions (Part I)

2.     There is a significant improvement in SCT scores when students progress from Year 3 to Year 4 within the clinical phase of their undergraduate medical program, as measured by retrospective analysis of pooled and matched (same cohort) SCT assessment scores for 5 cohorts of medical students (Part II)

## Methods

### Study design and participants

This study was a two-pronged practitioner inquiry within one medical school context in Australia.^20^ Part I of the study involved a cross-sectional study design. Three groups of participants took part in the study: final year medical students (N=105) completed the 40 items SCT as part of their invigilated written summative examination in October 2015; registrars in general practice training (N=19); and practising General Practitioners (N=13), completed the same SCT paper in January 2016. The registrars who were junior doctors with less than 4 years post-graduation clinical experience, were recruited via general email invitations distributed to School alumni. The General Practitioners (GPs) participants, who had at least 5 years of post-fellowship practice experience in General Practice, were also part-time Problem Based Learning (PBL) tutors at the School. Both groups of graduated doctors were volunteer participants in the study.

The expert reference panel (N = 17) comprised specialists in relevant disciplines who had provided answers to the SCT questions in the written paper, and their responses to each SCT item were used as the basis for the scoring of the responses by participants in the three study groups, using the classical weighted aggregate partial scoring approach.^3^

Part II of the study involved a retrospective analysis of pooled and matched summative SCT assessment scores for 5 cohorts of medical students (2012 to 2017). Expert reference panels (N=13-18) comprised specialists in the relevant disciplines and their responses were used as the basis for the scoring using the same approach as in Part I.

Ethics approval for the study was obtained from the University’s Human Research and Ethics Committee (#018161S). To ensure the confidentiality and anonymity of the participants, all data were de-identified prior to the commencement of data analysis.

### Data collection

In Part I of the study, a set of 40 SCT questions, based on 15 case scenarios covering the disciplines of Medicine, Surgery, Paediatrics, Obstetrics & Gynaecology, Psychiatry and General Practice, were developed to assess clinical reasoning according to the assessment blueprint of the medical programme. Each SCT question was reviewed by discipline-specific experts and the assessment academics at the School to ensure content validity. The usual format for construction of SCT items was employed.^12^^,^^21^ Special attention was made to ensure that there was a balance between items that attract extreme responses and those that attract median response options. As previously reported in the literature, careful balancing of item response options aimed to minimise the threats to validity by test-wise students who may try to game the examination, or the lowest quartile students trying to avoid extreme option answers.^22^^-^^24^ The set of 40 SCT questions were given to the 3 participant groups described above. Prior to this end-of-year summative examination (October 2015), all medical students had sat for a formative mid-year SCT examination and a practice online SCT quiz. The GP registrars, and experienced GPs completed the same set of SCT items in January 2016. Junior registrars and experienced GP study participants were given a detailed explanation of the structure and scoring of SCT as well as a sample set of SCT questions before they were asked to answer the set of SCT questions. After providing consent to participate, the junior registrars attempted the SCT online, using a survey template where all answers were collected anonymously. The experienced GP participants attempted the same set of SCT questions on campus under invigilation, using a paper-based format similar to the medical students.

Part II of the study involved retrospective analysis of the summative SCT scores for five cohorts of medical students in their clinical years from 2012 to 2017 inclusive. As part of the invigilated written end-of-year summative assessment at the School, all students completed a set of 40 SCT questions in their penultimate (Year 3) and final (Year 4) clinical year. As in Part I of the study, SCT scoring was based on the classical aggregated partial scoring method, with a full mark being awarded for concordance with the majority of the expert panel and a partial weighted score for concordance with the minority of the panel.^12^

### Data analysis

A one-way between-subjects ANOVA was conducted on the data from the first part of this study, to compare the difference in mean SCT scores obtained by senior medical students, GP registrars and practising GPs. For the comparison, p <.05 was considered statistically significant. In the second part of the study, pooled and matched SCT scores in Year 3 and Year 4 for individual students from five cohorts (2012 to 2017), were analysed for evidence of significant progression, or the lack thereof, using a repeated measure t-test. Each student’s Year 3 SCT score was paired and matched with their respective Year 4 scores when the student had progressed to the final year of their MBBS/MD program. The SPSS statistical package version 25 (IBM Corp., Armonk, NY) was used for the statistical analysis in both parts of the study.

## Results

The Cronbach’s alpha value for scores from each SCT paper was in the range of 0.62 to 0.86 (2012-2017), providing evidence of acceptable reliability (i.e. internal consistency) of the SCT scores. Part I of the study indicated a significant main effect of clinical experience on performance in the SCT, at the p<0.05 level for the three stages of medical career i.e. medical students, junior GP registrars, and, experienced GPs [F_(__2,136)_=6.215, p=0.003]. The effect size (Eta squared, η²=0.084) was moderate, based on Cohen's guidelines (small effect size: η²= 0.01; medium effect size - η²=0.06; large effect size - η²= 0.14).^25^^,^^26^

**Figure 1:**
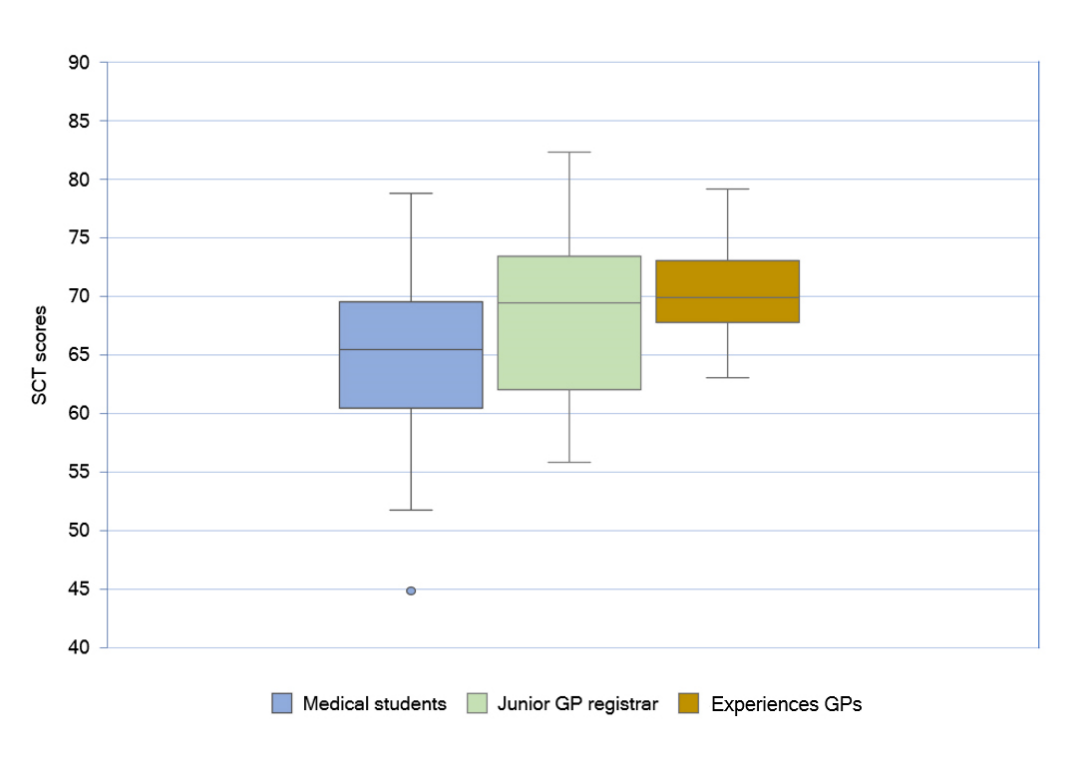
Comparison of SCT scores of medical students, junior registrars and practising GPs

Post hoc comparisons using the Bonferroni method indicated that the mean SCT score for experienced GPs (M = 70.39, SD=4.41, N=13) was significantly higher (p=0.011) than the mean SCT score of medical students (M= 64.90, SD = 6.30, N=105). However, the mean SCT score for junior GP registrars (M=68.36, SD=7.20, N=19) did not differ significantly from the mean SCT scores of medical students (p= 0.069) and the experienced GPs (p=1.000). The expert panel’s (N=17) average score was 79.40% (SD=10.8). The results are represented in the box plot (Figure 1).

**Figure 2:**
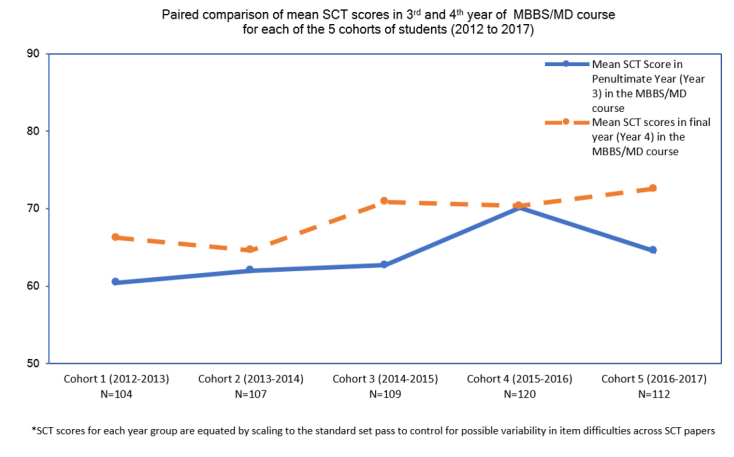
A paired comparison of mean SCT scores in Year 3 and Year 4 medical students for 5 cohorts (from 2012 to 2017)

Part II of the study compared pooled and matched data for five cohorts (2012-2017) of medical students’ SCT scores in the penultimate (Year 3) and final year (Year 4) of the undergraduate medical program. A repeated measure t-test indicated that the mean SCT scores for Year 4 students (M= 68.90, SD=7.79, N= 584) was higher than the mean SCT score for Year 3 students (M=64.03, SD=7.98, N=598). This difference in penultimate and final year students’ mean SCT score, was statistically significant [t_(__552)_=12.78, p< 0.001]. A medium effect size was observed in the data, with Cohen’s d repeated measures, pooled=0.544 (95%CI= 0.417 to 0.657). The means of SCT scores from 2012 to 2017 for the Year 3 and Year 4 students are represented in Figure 2.

## Discussion

This study has provided evidence for the construct validity of SCT scores as a measure of clinical reasoning ability of undergraduate medical students. When the same set of SCT questions were given to senior medical students, junior GP registrars and experienced GPs, a significant upward progression of the SCT scores, from senior medical student level (relative novices) to practising GP clinician level (experienced clinicians), was noted. This suggests that GPs have more well-developed clinical reasoning skills, supporting the earlier observation that SCT scores tend to consistently increase with increasing level of training.^17^ This result correlates with the study results showing progression of SCT scores from medical students to residency trainees in a Neurology training program.^1^

Although there was no statistical significance difference between the mean SCT scores for senior medical students and junior GP registrars, an upward trend was evident (from 64.90% to 68.36%). This could partially be explained by the fact that significant improvement in clinical reasoning with clinical experience is a progressive process occurs over a significant period of time. Subra et al. found that postgraduate students’ clinical reasoning skills take time to develop and the largest improvement occurs during the first 18 months of training in general practice.^19^ The smaller effect size could also be due to a plausible confounder – i.e. the medical students’ more recent, and better experience (with more practice), with the assessment modality, compared to either the registrars or experienced GP participants.

The second part of the study using paired data from 2012 to 2017 demonstrated progression in medical students’ SCT scores from Year 3 to Year 4. The effect size of 0.544 indicates that when medical students advance from Year 3 to Year 4, gaining more clinical experience, their mean SCT score also increases by 0.5 standard deviation.^27^ A contrasting result was reported from another Australian university with a 6-year undergraduate medical programme, where the SCT scores of Year 6 students in a formative assessment were compared to those of Year 5 students undertaking an end-of-year summative assessment.^6^ In this instance, Year 6 students had less experience in answering SCT format questions. The significantly lower SCT means scores achieved by Year 6 students compared to those in Year 5 may highlight the benefit of prior experience with SCT items, and the potentially positive effect of sitting a high-stakes examination on candidate performance.

The results from the current study suggest that clinical experience does have an effect on performance in SCT, providing further support for the construct validity of scores from this format of assessment. Whilst previous studies have reported similar results at post-graduate medical education stage and shown progression of scores as trainees advance through their training in Neonatology and Psychiatry, there have been no reports of progression of SCT scores when the same set of SCT items are used to compare the clinical reasoning ability of medical students, junior doctors and experienced clinicians.^8^^,^^18^ A study from Brazil has shown progression of SCT scores from students in the pre-clinical phase to those in the clinical phase (51.6% to 63.4%) using 10 clinical cases. However, the authors concluded that the implementation of this exam format is difficult in under-resourced institutions and have not followed up on these findings.^28^

### Limitations of the study

In the summative assessment program of our medical school, Script Concordance Testing is a subset of the written paper, which limits each SCT section to 40 items only. The multiple-choice and short answer questions aim to test student knowledge, and ability to apply knowledge to clinical scenarios, whilst the SCT questions are included to test clinical reasoning. Including a greater number of SCT questions may help to elucidate whether there is a significant difference between medical student and junior GP registrar performance, as well as whether there is a significant difference between performance of junior GP registrars and experienced GP clinicians. It should also be noted that the SCT assessment is a high stakes examination for medical student participants, in contrast to registrar and GP participants in this study, where there is absolutely no stake in their participation in answering the SCT questions. Unequal sample size for each group used for comparison in the first part of this study should also be acknowledged as a potential limitation. This is particularly so for the sample size of junior registrar participants, which may, to a certain extent, explains the observation that while the scores of junior registrars were higher than senior medical students, collectively the difference in mean scores has failed to reach statistical significance. Nevertheless, the one-way ANOVA statistic used, is rather robust for comparisons involving unequal sample size in groups.^29^ The findings are also limited in that the analysis was only performed on student results from one medical school.

More importantly, we acknowledge the fact that SCT scores are vulnerable to various validity threats and hence we are cautious not to over-claim with unrealistic inferences based on results from our limited data and simple convenient research design.^9^^,^^17^^,^^30^^,^^31^ Nonetheless the current study adds to the limited available literature examining the progression of SCT scores with advancing clinical experience, especially in the undergraduate medical education setting.

A further study is underway to investigate the addition of a “think-aloud” written explanation to each SCT clinical scenario where the candidates are asked to explain their reasoning for choosing a particular response for each SCT question.^32^^,^^33^ The response process validity of SCT scores as a measure of the clinical reasoning skills of undergraduate medical students would be enhanced if the majority of students chose the correct answer (to which the majority of experts agreed) for the correct reason, rather than providing correct answer-wrong reason responses. This qualitative data will add to the understandings of basis for any differences in SCT capability noted across the vertical continuum of medical education.

Further studies should look into the progression of clinical reasoning capabilities from Year 3 to Year 4 of the graduate-entry medical program in more than one medical school. A prospective longitudinal study involving a greater sample size of medical graduates would be more powerful in determining whether there is positive progression in clinical reasoning ability as measured by the SCT during junior doctor years, as compared with doctors doing fellowship training and subsequent clinical practice.

## Conclusions

The increase in SCT scores of experienced GPs compared to medical students, and the higher SCT scores of final year medical students compared to their student peers in the penultimate year, support previous research findings that SCT scores consistently increase with increasing level of training. This study in one context of undergraduate medical education added further evidence to the body of literature concerning the construct validity of SCT as an assessment modality. Prospective longitudinal studies with larger sample sizes are recommended to further test the construct validity of SCT scores.

### Acknowledgments

We would like to acknowledge Miss Eunice Lau for her support in collating the anonymous SCT examination data.

### Conflict of Interest

The authors declare that they have no conflict of interest.
